# Unexpected enzymatic function of an ancient nucleic acid-binding fold

**DOI:** 10.1093/nar/gkaf328

**Published:** 2025-04-25

**Authors:** Rylan R Watkins, Stella Bockelman, Anna Vradi, Kaylee Grabarkewitz, Alexa Pyun, Josephine Stark, Vicki H Wysocki, Juan D Alfonzo, Karin Musier-Forsyth

**Affiliations:** Department of Chemistry and Biochemistry, Center for RNA Biology, Ohio State University, Columbus, OH, 43220, United States; Department of Chemistry and Biochemistry, Center for RNA Biology, Ohio State University, Columbus, OH, 43220, United States; Department of Chemistry and Biochemistry, Center for RNA Biology, Ohio State University, Columbus, OH, 43220, United States; Department of Chemistry and Biochemistry, Center for RNA Biology, Ohio State University, Columbus, OH, 43220, United States; Department of Chemistry and Biochemistry, Center for RNA Biology, Ohio State University, Columbus, OH, 43220, United States; Department of Chemistry and Biochemistry, Center for RNA Biology, Ohio State University, Columbus, OH, 43220, United States; Department of Chemistry and Biochemistry, Center for RNA Biology, Ohio State University, Columbus, OH, 43220, United States; Department of Molecular Biology, Cell Biology and Biochemistry, The Brown RNA Center, Brown University, Providence, RI, 02912, United States; Department of Chemistry and Biochemistry, Center for RNA Biology, Ohio State University, Columbus, OH, 43220, United States

## Abstract

Aminoacyl-tRNA synthetases (ARSs) are indispensable for all living organisms and their associated aminoacyl–tRNA editing domains ensure the fidelity of translation. In eukaryotes, ARSs form a multi-aminoacyl–tRNA synthetase complex (MSC), which is assembled together with several nonsynthetase scaffolding proteins. The MSC found in *Trypanosoma brucei* (*Tb*) includes two proteins with oligosaccharide/oligonucleotide-binding (OB) folds—MSC-associated protein 1 (MCP1) and MCP2—and one known *trans*-editing factor, MCP3, an Ala–tRNA deacylase. The activity of MCP1 was unexplored until now. Our study shows that recombinantly-expressed and purified MCP1 also deacylates Ala–tRNAs despite lacking known tRNA-editing domain homology. Domain deletion studies reveal that the OB-fold houses the catalytic pocket and mutation of any one of three conserved OB-fold residues (K326, R331, S335) abolishes activity. Assays with *Saccharomyces cerevisiae* Arc1p reveal that MCP1’s deacylation activity is conserved across organisms. This discovery explains the 3′ CCA-end binding activity of this protein family and uncovers an ancient nucleic acid binding domain’s unexpected enzymatic function.

## Graphical Abstract

**Graphical Abstract:**
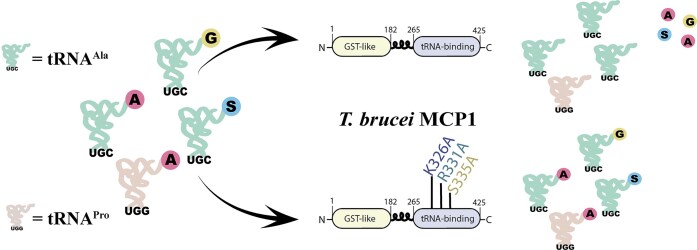

## Introduction

Aminoacyl–transfer RNA (tRNA) synthetases (ARSs) are an essential family of enzymes that catalyze the pairing of cognate amino acid and tRNA during protein synthesis. The aminoacylation reaction proceeds in two steps: (i) activation of the amino acid with ATP and (ii) transfer of the adenylated amino acid to the terminal adenosine (A_76_) of the tRNA [1]. Due to the similarity of 20 proteinogenic amino acids, ARSs can misactivate noncognate amino acids and misacylate the cognate tRNA [2–4]. As a result, organisms across all three domains of life encode editing mechanisms to maintain aminoacylation fidelity.

Aminoacyl–tRNA (aa–tRNA) proofreading occurs pre- or post-transfer of the activated amino acid. Pre-transfer editing refers to hydrolysis of the aminoacyl-adenylate (aa-AMP) [3, 5], whereas a mischarged tRNA can be cleared by one of two post-transfer editing mechanisms: *cis* or *trans*. In *cis*-editing, the aa–tRNA is never released from the ARS but the 3′-CCA end is translocated to an appended proofreading domain for deacylation. *Trans*-editing occurs following the release of a mischarged tRNA, which is then re-bound by the ARS editing domain or by a freestanding proofreading factor [3, 6]. Alanyl-tRNA synthetase (AlaRS) and prolyl-tRNA synthetase (ProRS) misactivate amino acids similar in size to cognate Ala (Gly and Ser) and Pro (Ala and Cys), respectively [7–14], and are examples of ARSs with appended editing domains. In addition, freestanding proteins homologous to the AlaRS and ProRS editing domains are ubiquitously expressed in all domains of life to keep the synthetases in check [3, 4, 6].

Many eukaryotes organize a subset of their ARSs into large multi-aminoacyl–tRNA synthetase complexes (MSCs) [15–18]. Nonsynthetase factors believed to act as scaffolds, as well as *trans*-editing proteins, are also present in these complexes; however, our understanding of the functional significance of these complexes and their associated components is limited. Even the smallest MSC reported to date, which is found in *Saccharomyces cerevisiae* (Sc), includes one non-ARS factor: Arc1p. The two other components of this MSC are methionyl-tRNA synthetase (MetRS) and glutamyl-tRNA synthetase (GluRS) [19]. Arc1p consists of an N-terminal GST-like domain and a C-terminal tRNA-binding domain (TRBD) that contains an oligonucleotide/oligosaccharide-binding (OB) fold with structural homology to *Aquifex aeolicus* (*Ae*) Trbp111. The N- and C-terminal domains of Arc1p are connected by a lysine-rich α-helical linker that is indispensable for RNA-binding [20–24]. The GST-like domain of Arc1p interacts with both MetRS and GluRS and is essential for anchoring both ARSs in the cytoplasm [20, 25, 26]. Several studies also show that the aminoacylation activity of MetRS and GluRS is stimulated by the presence of Arc1p *in vitro* [22, 27]. Although MSCs vary in composition between organisms, all have Arc1p homologs or paralogs, including those present in *Trypanosoma brucei* (*Tb*), *Arabidopsis thaliana*, and *Homo sapiens* [19, 28–30].

The current model of the *T. brucei* MSC consists of six ARSs—AlaRS, ProRS, MetRS, glutaminyl-tRNA synthetase (GlnRS), tryptophanyl-tRNA synthetase (TrpRS), and aspartyl-tRNA synthetase (AspRS)—and three non-ARS protein factors: MSC-associated protein 1 (MCP1), MCP2, and MCP3 [29]. Both MCP1 and MCP2 encode an Arc1p-like TRBD with an internal OB-fold [29]. Among the three MCP factors, MCP1 is the closest homolog to Arc1p as it also encodes an N-terminal GST-like domain tethered to the TRBD via a lysine-rich α-helical linker region. MCP2 has been shown to enhance MetRS aminoacylation *in vitro*, although the underlying mechanism of this activity is unknown. *Tb* MCP3 is a member of the INS superfamily of editing domains, named after the founding member, the bacterial ProRS “insertion” domain [3]. We recently showed that MCP3 is an Ala–tRNA deacylase that lacked tRNA discrimination *in vitro* and deacylated Ala–tRNA^Pro^ as well as cognate Ala–tRNA^Ala^ [31]. This promiscuous activity was significantly reduced in the presence of MSC factor AlaRS, with a more modest reduction observed in the presence of ProRS. Thus, we proposed that an important function of the *Tb* MSC may be to prevent Ala–tRNA^Ala^ deacylation by MCP3. The impact of non-ARS factors on MCP3 deacylation has not yet been investigated.

In this study, we successfully recombinantly expressed and purified *Tb* MCP1 and investigated its influence on MCP3-mediated Ala–tRNA deacylation *in vitro*. The presence of MCP1 significantly enhanced the single-turnover rate constant for substrate hydrolysis by MCP3. Unexpectedly, MCP1 alone exhibited deacylation activity, despite not being homologous to any known tRNA-editing domain. We discovered that the MCP1 OB-fold is crucial for this catalytic activity, consistent with recent X-ray crystallography studies revealing that *Ae* Trbp111 binds to the 3′-end of tRNA [24]. *Sc* Arc1p, previously thought to serve exclusively as a scaffold protein, showed the same catalytic function. This work advances our understanding of a conserved eukaryotic MSC-associated factor and reveals the enzymatic role of an ancient nucleic acid-binding fold.

## Materials and methods

### Recombinant protein expression and purification

Coding sequences for proteins of interest were obtained from the Kinetoplastid Informatics Resource (TriTryp) and the Saccharomyces Genomic Database (SGD). The TriTryp identifiers for *Tb* MCP1, Tb MCP2, and Tb AlaRS are Tb927.7.2400, Tb927.8.5330, and Tb927.6.700, respectively. The SGD identifier for the Sc Arc1p gene (ARC1) is S000003073. Sequences were codon optimized for expression in *Escherichia coli*, commercially synthesized, and cloned into pET15b (Arc1p, MCP1, MCP2) or pGS21a (AlaRS) bacterial expression vectors by GenScript technologies. pET15b N-His_6_*Tb* MCP1 (and all domain/point mutants), *Tb* MCP2, Sc Arc1p, and pGS21a *Tb* AlaRS (N-His_6_-GST) were transformed into Rosetta (DE3) *E. coli* (Novagen) via electroporation for expression. *Escherichia coli* cells expressing the protein of interest were grown in 2× YT media for *Tb* Arc1p, MCP1, and MCP2 (0.5–2 l), or terrific broth for *Tb* AlaRS, with antibiotics (100 μg/ml ampicillin, 30 μg/ml chloramphenicol) at 37°C with shaking (220 RPM) until the OD_600_ was between 0.5 and 0.8. Flasks were chilled on ice for 15 min prior to addition of isopropyl-β-D-1-thiogalactopyranoside (IPTG) to a final concentration of 0.2 mM. Proteins were induced overnight (16–18 h) at 18°C. Wild-type (WT) and mutant MCP1 constructs, Sc Arc1p, and Tb AlaRS were purified using Buffer A {0.1 M 3-morpholinopropane-1-sulfonic acid (MOPS), pH 7.6, 1 M NaCl, 10% v/v glycerol, 20 mM β-mercaptoethanol (BME), 1 mM tris(2-carboxyethyl)-phosphine (TCEP), 5 mM imidazole, 1 mM MgCl_2_, and 0.1% 3-[(3-cholamidopropyl) dimethylammonio]-1-propanesulfonate (CHAPS)}. Tb MCP2 was purified using Buffer B (0.1 M Tris–HCl, pH 8.5, 1 M NaCl, 10% v/v glycerol, 50 mM L-arginine, 20 mM BME, 5 mM imidazole, 1 mM MgCl_2_, and 0.1% CHAPS. Induced cells were pelleted at 6000 × *g* for 10 min at 4°C and then resuspended in pre-chilled isolation Buffer A or B at a ratio of 10 ml per gram of cell pellet. One cOmplete ethylenediaminetetraacetic acid (EDTA)-free protease inhibitor tablet (Roche) and 50 mg of lysozyme powder (Sigma) was added per 50 ml of cell suspension. Cells were continually stirred for 30 min on ice with 1% v/v Tween-20 (Bio-Rad) prior to a sonication cycle of 15 s on, 45 s off, at 50% amplitude for a total of 16 pulses using a microtip. Crude lysate was clarified at 20 000 × *g* for 30 min at 4°C. The soluble fraction was loaded onto a gravity flow column packed with Pierce EDTA-compatible high-capacity nickel resin (Thermo Fisher) equilibrated with isolation Buffer A or B. Proteins were eluted with an imidazole step gradient (20–500 mM) and analyzed by denaturing polyacrylamide gel electrophoresis [sodium dodecyl sulfate–polyacrylamide gel electrophoresis (SDS–PAGE)]. Peak protein fractions were pooled, concentrated, and buffer exchanged three times with 2× storage buffer using Amicon-Ultra molecular-weight cutoff (MWCO) centrifugal filters (Sigma). All proteins except MCP2 were exchanged into 0.1 M MOPS pH 7.6, 1 M NaCl, 10% w/v D-trehalose, 5 mM TCEP, and 0.1% CHAPS before being mixed with an equal volume of 80% v/v glycerol and long-term storage at −20°C at a final concentration of ∼100–300 μM. MCP2 was concentrated and exchanged into 0.1 M Tris–HCl, pH 8.5, 1 M NaCl, 0.1 M L-arginine, 2 mM TCEP, and 20% w/v D-trehalose before being mixed with an equal volume of 80% glycerol to obtain a final concentration of 50–100 μM. MCP2 was aliquoted and stored at −80°C to prevent aggregation. Optimal enzymatic activity for MCP1 was achieved when cell lysis, affinity capture, and storage was completed within a 12-h window. Protein concentrations were determined by measuring the absorbance at 280 nm using extinction coefficients obtained from Expasy ProtParam [32].

### Site-directed mutagenesis

MCP1 domain truncations and point mutations were generated by performing site-directed ligase-independent mutagenesis (SLIM) using the pET15b vector encoding the codon-optimized *Tb* MCP1 gene according to published protocols [33].

### *In vitro* transcription of tRNA

DNA templates for *in vitro* transcription (IVT) by T7 RNA polymerase were prepared by PCR from pUC19 plasmids containing tRNA genes of interest using Phusion high-fidelity DNA polymerase [New England Biolabs (NEB)]. DNA amplicons were purified using a PCR clean-up kit (Qiagen) and added to a 1-ml transcription reaction to a final concentration of 15 ng/μl. tRNAs were transcribed overnight at 25°C in a buffer consisting of 80 mM HEPES-K, pH 8.0, 30 mM Mg(OAc)_2_, 10 mM dithiothreitol (Research Products International), 5 mM spermidine, 0.01% Triton-X-100 (Thermo Fisher), 2% v/v PEG8000, 4 mM rNTPs (Thermo Fisher), 5 U inorganic yeast pyrophosphatase (Sigma), and recombinant P266L T7 RNA polymerase prepared in house as described [34]. IVT reactions were terminated with 50 mM EDTA, pH 8.0, and purified by denaturing polyacrylamide gel electrophoresis according to published protocols [35]. Briefly, reactions were mixed 1:1 with 2× RNA loading dye (89 mM Tris-Borate-EDTA, pH 8.3, 90% formamide, 20 mM EDTA, pH 8.0, 0.05% sodium dodecyl sulfate, 0.01% w/v bromophenol blue/xylene cyanol), loaded onto a 12% polyacrylamide gel containing 8 M urea, and electrophoresed in 1× Tris–Borate–EDTA, pH 8.3. tRNA bands were visualized by ultraviolet (UV)-shadowing, excised, crushed, and then soaked overnight in elution buffer (0.5 M NH_4_OAc, 2 mM EDTA, pH 8.0). Eluted tRNAs were recovered with a 3 kDa MWCO Amicon-Ultra centrifugal filter device, ethanol precipitated, and resuspended in milliQ-H_2_O before determining the concentration by measuring A_260_ on a NanoDrop ONE^c^ (Thermo Fisher) spectrophotometer (ϵ_260_ = 604 000 M^−1^cm^−1^).

### aa–tRNA substrate preparation

*In vitro* transcribed tRNA^Pro^ and tRNA^Ala^ (100 pmol) were 3′-end labeled with ^32^P via nucleotide exchange of the terminal adenosine (A_76_) using [^32^P]-α-ATP (Revvity) and *E. coli* tRNA nucleotidyltransferase (*Ec* NTase) according to published protocols [36]. Briefly, 100 pmol of tRNA was renatured by heating at 80°C for 2 min, 60°C for 2 min, and slow cool to room temperature following addition of 10 mM MgCl_2_. The refolded tRNA was added to a mix containing 0.1 M glycine, pH 9.0, 20 mM MgCl_2_, 0.1 mg/ml bovine serum albumin, 250 nM CTP, 50 μM sodium pyrophosphate, 50 μCi [^32^P]-α-ATP, and ∼0.5 mg/ml *Ec* NTase at 37°C for 5 min to remove the endogenous A_76_. The nucleotide exchange reaction was initiated by addition of 0.5 U of yeast inorganic pyrophosphatase (NEB) and allowed to proceed for 5 min before termination with 3× volume of 0.2 M NaOAc, pH 5.1. [^32^P]–tRNA was recovered by extraction with acidic phenol:chloroform, pH 4.5 (Ambion) and ethanol precipitation in the presence of 30 μg GlycoBlue (Thermo Fisher).

Prior to aminoacylation reactions, the ethanol precipitated [^32^P]–tRNA was resuspended with 3000 pmol (30 μl of 100 μM) nonradiolabeled tRNA in 10 mM HEPES-KOH, pH 7.0. This tRNA mix was renatured by heating at 80°C for 2 min, 60°C for 2 min, followed by addition of 10 mM MgCl_2_ and slow cooling to room temperature for 5 min. A portion (∼100 pmol at 5 μM) of folded [^32^P]-tRNA was reserved for use as a concentration standard for thin-layer chromatography (TLC) analyses described below. The remaining [^32^P]-tRNA was (mis)aminoacylated using *Tb* AlaRS (Ala–, Gly–, Ser–tRNA^Ala^) or *Tb* ProRS (Abu–, Ala–, Cys–, Pro–tRNA^Pro^) in a buffer containing 0.1 M Tris–HCl, pH 8.0, 30 mM KCl, 20 mM MgCl_2_, 0.1 mg/ml bovine serum albumin (BSA), 10 mM ATP, 2 mM TCEP, 0.5 U yeast inorganic pyrophosphatase (NEB), and the following amino acid concentrations: 10 mM L-Ala (AlaRS charging), 400 mM L-Ala (ProRS mischarging), 10 mM L-Pro, 400 mM L-Ser, 400 mM Gly, 400 mM Abu, or 10 mM L-Cys. Reactions were incubated for 30 min at 37°C, with the exception of Cys-tRNA^Pro^ mischarging, which was performed for 2 h. aa–tRNAs were recovered by extraction with acidic phenol:chloroform, pH 4.5 (Ambion), followed by ethanol precipitation and resuspension in 5 mM NaOAc, pH 5.1 before storage at −80°C.

The concentration of aa–tRNA was determined by incubating 2 μl of recovered [^32^P]-aa–tRNA and 2 μl of a 5 μM [^32^P]-tRNA standard in 8 μl of digestion buffer [250 mM NaOAc, pH 5.1, 280 mM NaCl, 4.5 mM ZnSO_4_, 0.5 U/μl of S1 nuclease (Promega)]. Digestions (2 μl) were spotted on a polyethyleneimine (PEI) TLC plate (Sigma) and products ([^32^P]-AMP and [^32^P]-aa-AMP) were resolved using a mobile phase containing 5% v/v acetic acid and 2 g/L NH_4_Cl. TLC plates were dried and exposed to phosphorimaging screens (Cytiva) overnight and imaged on a Typhoon RGB phosphorimager. Densitometry was performed using ImageJ or ImageQuant (Cytiva). The radiometric densities determined for the [^32^P]-aa–tRNAs were normalized to the values obtained for the 5 μM [^32^P]–tRNA standard to estimate concentration.

### Deacylation assays

Deacylation assays were performed under single-turnover conditions with 500 nM of protein and 50 nM of aa–tRNA in a reaction buffer consisting of 0.1 M HEPES-KOH, pH 7.9, 20 mM KCl, 5 mM MgCl_2_, 1 mM TCEP, 0.1 mg/ml BSA, and 0.05% Tween-20 as previously described [31]. Briefly, a 2× enzyme mix and a 2× aa–tRNA mix were prepared separately: enzymes were diluted to 1 μM with 2× reaction buffer and aa–tRNA was diluted to 100 nM with milli-Q water. Both 2× mixes were incubated at 27°C for at least 2 min before initiating the reaction by mixing equal volumes of 2× enzyme and 2× aa–tRNA. At desired time points, 2 μl of the reaction was added to 8 μl of digestion buffer and incubated at room temperature for 30 min. S1 nuclease digestion products were analyzed by PEI-TLC and imaged as described above. ImageQuant software (Cytiva) was used to obtain the densitometric ratio of charged to uncharged tRNA as a function of time. Data were fit to a single-exponential decay equation (with a plateau constrained to zero) using GraphPad Prism to obtain *k_obs_* values.

### Active-site titration assays

The concentration of purified recombinant His_6_-GST-AlaRS was determined by active-site titration as previously described [37, 38]. Briefly, 2.5 μM of His_6_-GST-AlaRS (as determined by UV_280_ and ϵ_280_= 148 630 M^−1^ cm^−1^ estimated by Expasy ProtParam [32]) was mixed with 50 mM HEPES-KOH, pH 7.5, 20 mM KCl, 10 mM MgCl_2_, 0.1 mg/ml BSA, 2 mM TCEP, 0.5 U yeast inorganic pyrophosphatase (NEB), and 5 mM L-Ala. The reaction was initiated by addition of nonradiolabeled ATP spiked with 5 μCi of [^32^P]-α-ATP (Revvity) to a final concentration of 5 μM. ATP consumption was monitored over time by quenching 2 μl of the reaction into 8 μl of 0.2 M NaOAc, pH 5.1. The amount of active AlaRS was determined by spotting 1 μl of quenched reaction products on a PEI-TLC plate, resolving them in a mobile phase of 4 M urea, 0.75 M KH_2_PO_4_, pH 3.5, followed by phosphorimaging and densitometry analyses.

### Aminoacylation kinetic assays

Aminoacylation kinetic assays were performed using 50 nM AlaRS in the absence and presence of 4 μM MCP1. Assays were carried out in the deacylation reaction buffer described above, supplemented with 4 mM ATP, saturating amino acid (1 mM L-Ala, or 330 mM Gly/L-Ser), 4.5 μM nonradiolabeled tRNAs (Ala, Pro, Met, Gln), and trace [^32^P]–tRNA^Ala^ (< 10 pmol). Reactions were conducted at 27°C in a final reaction volume of 16 μl. At desired time points, 2 μl of the reaction was quenched into 8 μl of digestion buffer. Reaction products were analyzed by PEI-TLC as described above. The ratio of aminoacylated to uncharged tRNA^Ala^ was plotted against time and fit to the Michaelis-Menten equation with GraphPad prism.

### Mass photometry

Mass photometry (MP) experiments were performed on a Refeyn Two^MP^ instrument and analyzed using Refeyn Aquire^MP^ software. Glass slides were cleaned by washing with alternating milli-Q water and 100% isopropanol (two rinses each). The slides were dried under a stream of nitrogen (% purity). Gaskets were cleaned similarly but were allowed to air dry for at least 30 min after removal from the nitrogen stream. Prior to each experiment, a clean gasket was placed in the center of a clean glass slide and put into the slide holder. MP reaction buffer (19.5 μl of 50 mM HEPES-KOH, pH 7.6, 50 mM NaCl, 2 mM MgCl_2_, 0.5 mM TCEP) was added to gasket well and focus was found for the glass slide in the detector region using the acquisition software. The instrument was calibrated daily using a mixture of thyroglobulin (monomer and dimer species; Sigma T91451VL) and β-amylase (monomer, dimer, and tetramer species; Sigma A87811VL) with known molecular weights. This mixture was added to the reaction buffer already present in gasket well #1 such that the final concentration for both proteins was 12.5 nM. A 1-min movie was acquired and the measured ratiometric contrast values were related to the corresponding molecular weights using Refeyn Discover^MP^ software. Stock aliquots were made for WT MCP1 and ΔGST MCP1 by diluting from storage buffer into MP reaction buffer to final concentration of 10 μM. The tRNA^Ala^ was refolded at 10 μM in 50 mM HEPES, pH 7.6, by first incubating at 80°C for two min, followed by 60°C for 2 min, addition of 2.5 mM MgCl_2,_ and incubation on ice for >30 min. Reactions containing MCP1 or ΔGST MCP1 alone were diluted to 2 μM in MP reaction buffer and incubated at room temperature for 10 min before being placed on ice. For reactions in the presence of tRNA, RNA–protein complex was formed at a ratio of 1:2 (1 μM tRNA^Ala^ : 2 μM MCP1) and incubated at room temperature for 10 min before being placed on ice until data collection. Data was acquired as described above: 19.5 μl of MP reaction buffer was added to an empty gasket well and focus was found. Samples (WT MCP1, ΔGST MCP1, or tRNA^Ala^ : MCP1 complex) were diluted to achieve a final protein concentration of 50 nM in MP reaction buffer, mixed thoroughly in the well, and a 1 min movie was immediately acquired. Each experiment was carried out in triplicate and all data analysis was performed with Refeyn Discover^MP^ software.

## Results

### *Tb* MCP1 displays ala-tRNA deacylation activity

**Figure 1.**
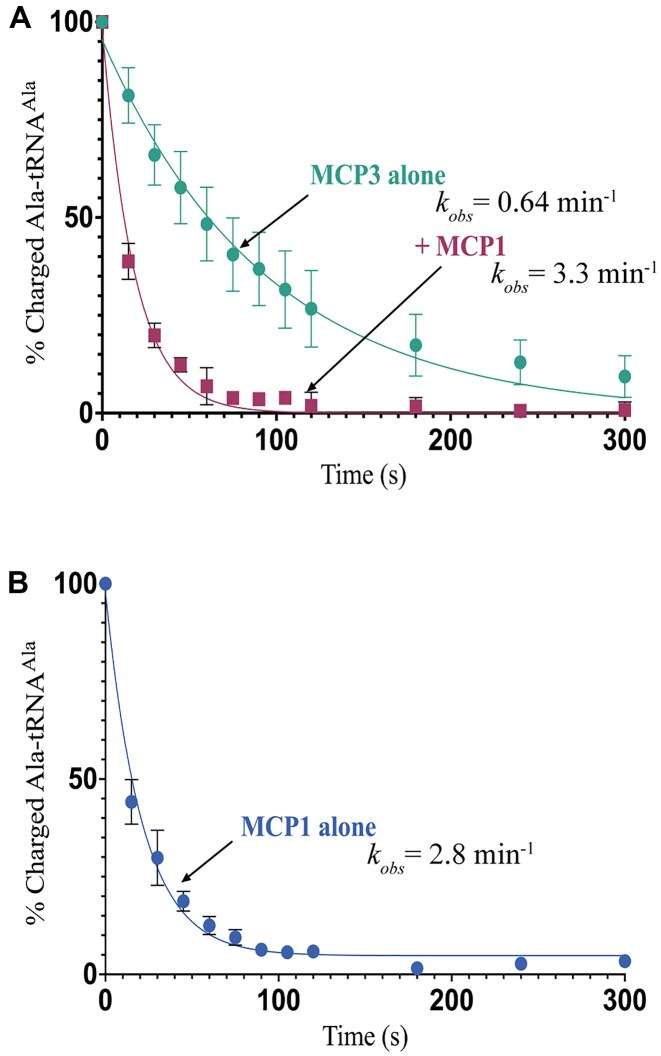
Ala–tRNA^Ala^ deacylation by *Tb* MCP1. (**A**) Single-turnover deacylation of 50 nM Ala–tRNA^Ala^ by 500 nM *Tb* MCP3 in the presence (▪) and absence (•) of 500 nM MCP1. The results for MCP3 alone are from ref [31]. (**B**) Single-turnover deacylation of 50 nM Ala–tRNA^Ala^ by 500 nM of *Tb* MCP1 alone. Assays were conducted at 13.5°C. Error bars represent the standard deviation of three independent trials.

To test the effect of *Tb* MCP1, a non-ARS MSC member, on deacylation by *Tb* MCP3, the two proteins were pre-incubated prior to addition of pre-formed Ala–tRNA^Ala^. Time-course assays were performed at 13.5°C; this temperature allowed measurement of MCP3 activity, which is very rapid at physiological temperature. *Tb* MCP1 appeared to enhance the single-turnover rate constant (*k*_obs_) for MCP3-catalyzed Ala–tRNA^Ala^ hydrolysis by ∼5-fold to ∼3.3 min^−1^*versus* ∼0.64 min^−1^ in the absence of an added factor (Fig. 1A). Based on this result, we next measured the Ala–tRNA^Ala^ deacylation activity of MCP1 alone. Surprisingly, we observed robust hydrolysis of Ala–tRNA^Ala^ (*k*_obs_ ∼2.8 min^−1^) (Fig. 1B). Thus, our initial conclusion that MCP1 stimulates the activity of MCP3 is likely not correct; the *k*_obs_ reported in Fig. 1A (3.3 min^−1^) results from the combined effect of catalysis by both factors. This result was unexpected, as MCP1 is an *Sc* Arc1p homolog and lacks significant sequence similarity to MCP3 or any known family of tRNA editing domains.

### TRBD is essential for ala-tRNA deacylation by *Tb* MCP1

**Figure 2.**
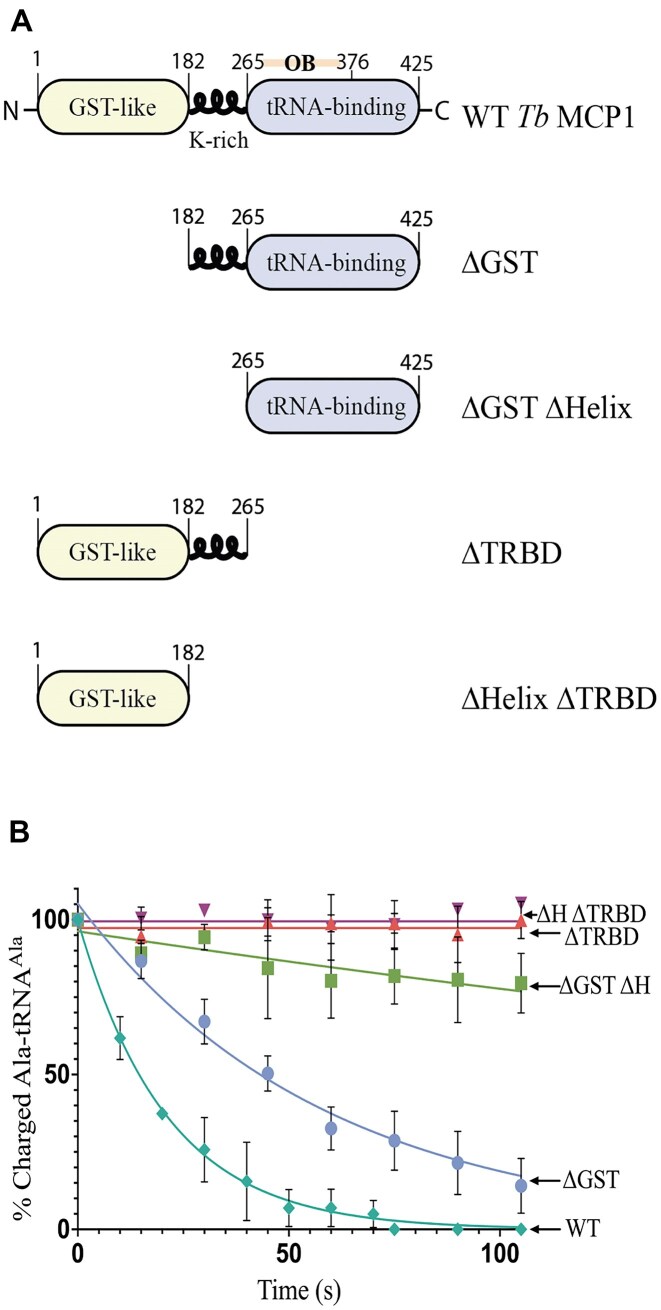
Schemes and deacylation activity of *Tb* MCP1 domain mutants. (**A**) Schemes of the recombinantly expressed and purified WT MCP1 protein and truncation mutants. (**B**) Single-turnover kinetic analysis of WT MCP1 and domain mutants. Deacylations were conducted at 27°C with 50 nM of Ala–tRNA^Ala^ and 500 nM of protein. ΔH refers to helix deletion as shown in the scheme in panel (A). Error bars represent the standard deviation of three independent trials. Rate constants for each mutant are reported in Table 1.

To determine the domain of MCP1 responsible for catalytic activity, we recombinantly expressed and purified the *Tb* MCP1 domain truncation variants shown in Fig. 2A. SDS–PAGE analysis of the MCP1 constructs showed that each protein is >95% pure (Supplemental Fig. S1). We tested the *trans*-editing activity of each mutant by monitoring deacylation of pre-formed Ala–tRNA^Ala^ over time at the physiological temperature for insect-stage (procyclic) *Tb* parasites (27°C) [39]. Removal of the TRBD abolished editing, while deletion of the GST-like and lysine-rich linker domains reduced the *k*_obs_ ∼3-fold and ∼21-fold, respectively (Fig. 2B and Table 1).

**Table 1.** Single-turnover rate constants for the deacylation of Ala–tRNA^Ala^ by WT *Tb* MCP1 and domain mutants

MCP1 protein	*k*_obs_ (min^−1^)^a^	Fold-change^b^
**WT**	2.88 ± 0.735	-
**ΔGST**	1.04 ± 0.216	2.77
**ΔGST ΔH**	0.136 ± 0.096	21.2
**ΔTRBD**	N.D.	>100
**ΔH ΔTRBD**	N.D.	>100

^a^Rate constants are an average of three independent trials conducted with 500 nM of enzyme and 50 nM of Ala–tRNA^Ala^ at 27°C. Reported error represents the standard deviation between trials.

^b^Fold-change is relative to WT activity.

### OB-fold residues Lys326, Arg331, and Ser335 are critical for deacylation

**Figure 3.**
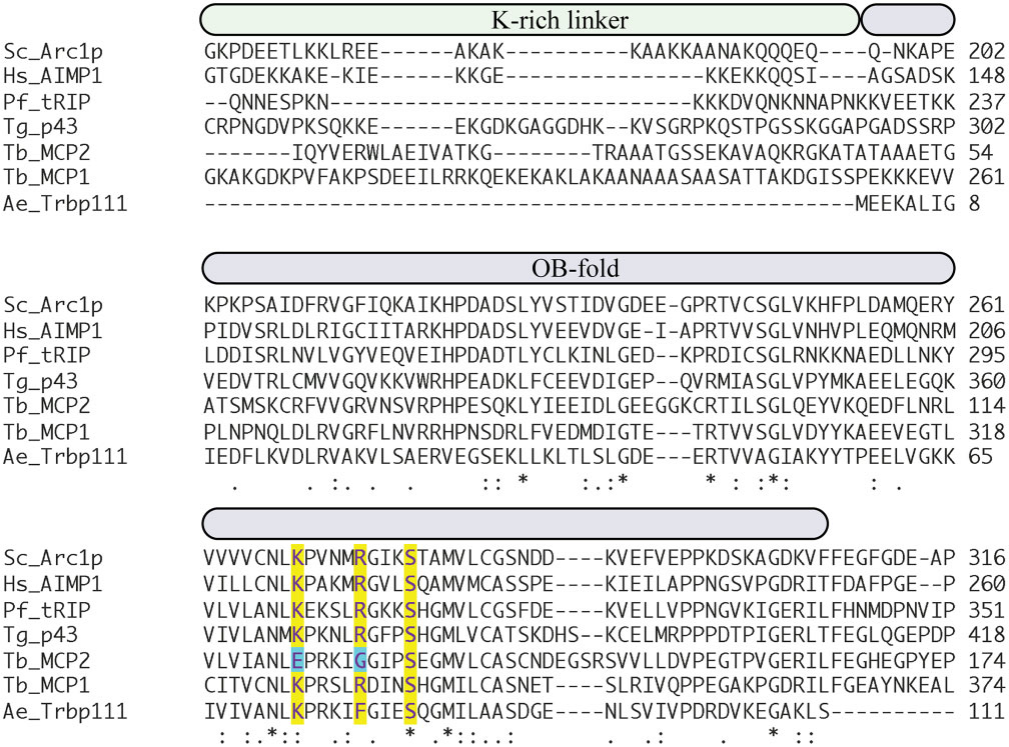
Multiple sequence alignment of OB fold region of MCP1-like proteins. Primary amino acid sequences are from NCBI BLAST and aligned using EMBL Clustal Omega [42]. The sequences shown are for the OB-fold portion of the TRBD. Residues highlighted in yellow were mutated in this work in the context of *Tb* MCP1. Residues marked by (∗), (:), or (.) correspond to strictly conserved, highly similar, or moderately similar, respectively. The *Tb* MCP2 residues highlighted in blue are discussed in the text. *Sc = Saccharomyces cerevisiae; Hs = Homo sapiens; Pf = Plasmodium falciparum; Tb = Trypanosoma brucei; Tg = Toxoplasma gondii; Ae = Aquifex aeolicus*.

**Figure 4.**
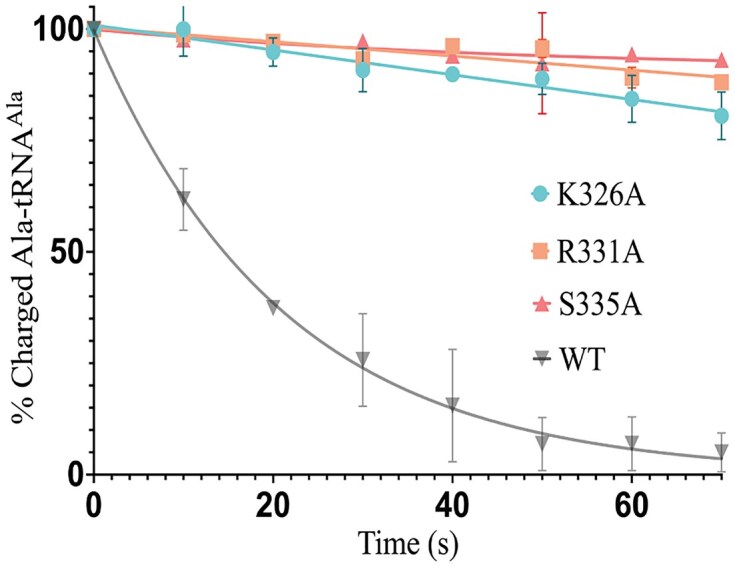
Deacylation activity of WT *Tb* MCP1 and site-directed mutants. Single-turnover deacylation of 50 nM Ala–tRNA^Ala^ by 500 nM of WT (*n* = 3) or MCP1 point mutants (*n* = 2) at 27°C. Error bars represent the standard deviation of two or three independent trials.

A recent X-ray crystallography structure of *E. coli* (*Ec*) tRNA^Ile^ bound to *Ae* Trbp111, a standalone homolog of the MCP1 OB-fold, revealed that binding occurred to the 3′ CCA-end of the tRNA [24]. Studies with the homologous *Sc* Arc1p showed K269, R274, and S278 were major contributors to tRNA binding—mutation of R274 (R274A) had the largest impact and resulted in a ∼9-fold decrease in binding affinity [24]. These residues align with residues in *Ae* Trbp111 that interact with the tRNA CCA-end and correspond to K326, R331, and S335 in *Tb* MCP1. They are highly conserved in other MCP1-like OB-fold proteins in humans, *Plasmodium falciparum (Pf)*, and *Toxoplasma gondii (Tg)* (Fig. 3). We mutated these positions in *Tb* MCP1 to Ala and measured the impact on enzymatic activity. Our results showed that each point mutation abolished Ala–tRNA^Ala^ deacylation (Fig. 4). Based on the published Arc1p/Trbp111 model, K326 is predicted to interact with the N1-amine of A_76_, R331 forms a cation–$\pi$ interaction with the C75 nucleobase, and S335 interacts with the nucleobase of A_76_ [24]. The INS superfamily of editing factors, with one exception, use a conserved Lys to position the terminal adenosine into the active site for deacylation [3, 40, 41]. We hypothesize that K326 or R331 may contribute to this function in MCP1.

### The GST domain and tRNA contribute to homodimerization of MCP1

Recent analytical ultracentrifugation (AUC) data suggests Sc Arc1p associates with tRNA with 2:1 stoichiometry, but in the absence of tRNA, the population of protein is predominantly monomeric [24]. The MCP1/Arc1p homologs and MSC scaffolding factors in other protists, *Pf* tRIP (tRNA import protein) and *Tg* p43, are reported to be homodimeric [43, 44]. The oligomeric state of *Tb* MCP1 has not yet been investigated.

**Figure 5.**
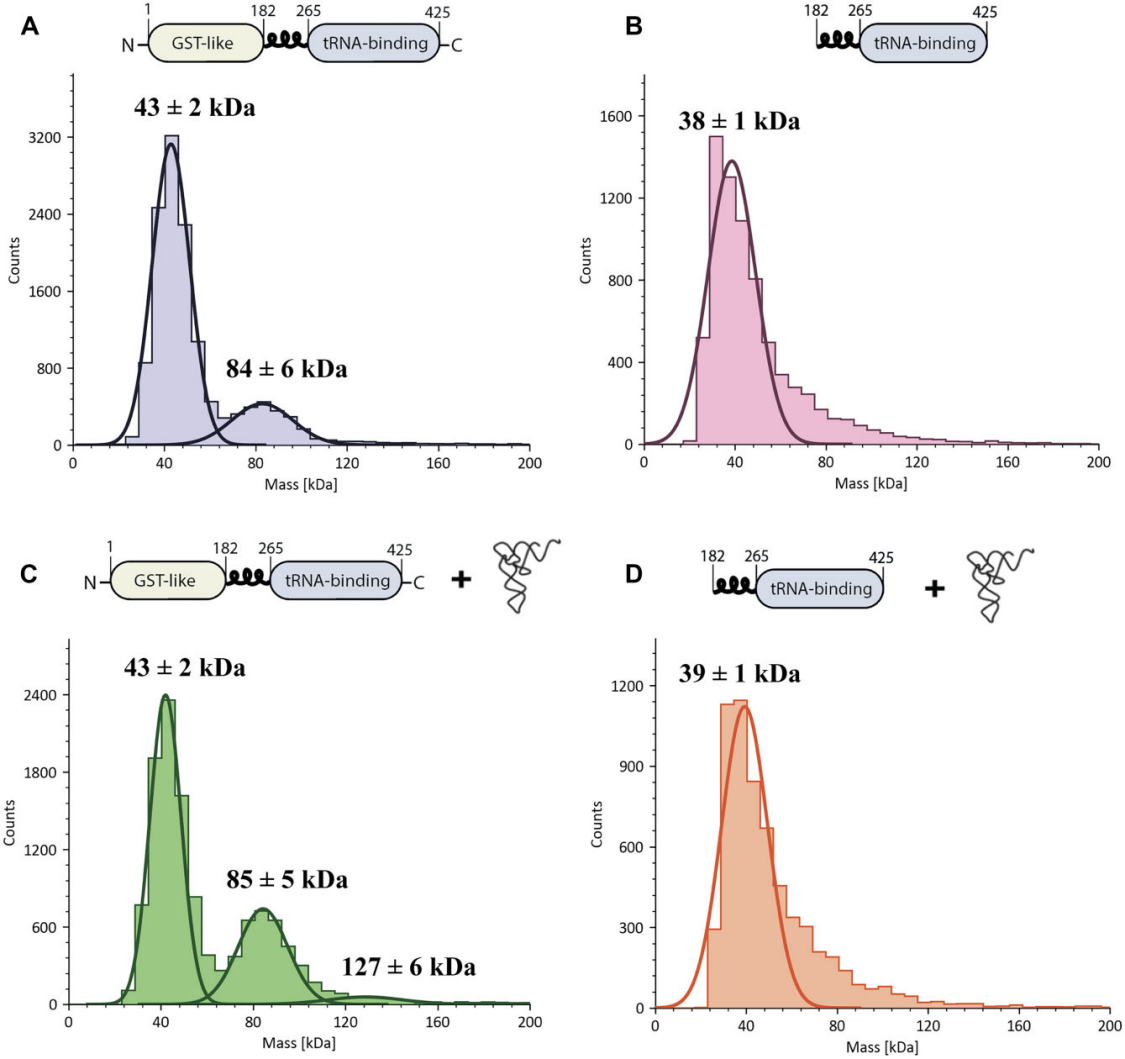
MP analysis of WT and ΔGST MCP1. MP analysis of (**A**) 50 nM WT MCP1, (**B**) 50 nM ΔGST MCP1, (**C**) 50 nM WT MCP1 + 25 nM tRNA^Ala^, and (**D**) 50 nM ΔGST + 25 nM tRNA^Ala^. Histograms represent the average of three independent trials.

MP is a solution-phase mass measurement technique that is compatible with most buffers and can be used to determine oligomerization states and binding stoichiometries [45–47]. Using MP, we first tested the oligomeric state of *Tb* MCP1 alone at 50 nM—sample concentrations above this will oversaturate the light scattering detector. Recombinant WT His_6_-MCP1 has a theoretical molar mass of ∼48 kDa—MP detected two distinct binding events with molar masses of ∼43 kDa and ∼84 kDa (Fig. 5A). These data suggest that in the absence of tRNA, WT *Tb* MCP1 is predominantly a monomer (85%), while only ∼17% of the protein population is dimeric under these conditions. A single peak was observed for the ΔGST MCP1 mutant corresponding to a ∼38 kDa species, which is larger than the theoretical monomeric mass (∼28 kDa) (Fig. 5B). MP is more accurate for measuring the mass of molecules >40 kDa, which may explain the overestimation of the monomeric ΔGST MCP1 molar mass. Nevertheless, these data suggest the GST domain contributes to dimerization.

We next examined the complex between *Tb* MCP1 and tRNA^Ala^ by MP. Two major and one minor mass distributions were observed: 43 kDa (MCP1 monomer), 85 kDa (MCP1 dimer), and 127 kDa (minor species) (Fig. 5C). In the presence of tRNA, the 85 kDa dimer peak represented ∼31% of the species, suggesting that the presence of tRNA shifts the equilibrium toward dimer. Although very little complex was observed under these conditions, the 127 kDa population is consistent with a 2:1 MCP1:tRNA^Ala^ complex (∼121 kDa). The addition of tRNA to the ΔGST variant did not change the oligomeric state (Fig. 5D).

### OB-fold catalytic pocket accommodates small amino acids

**Figure 6.**
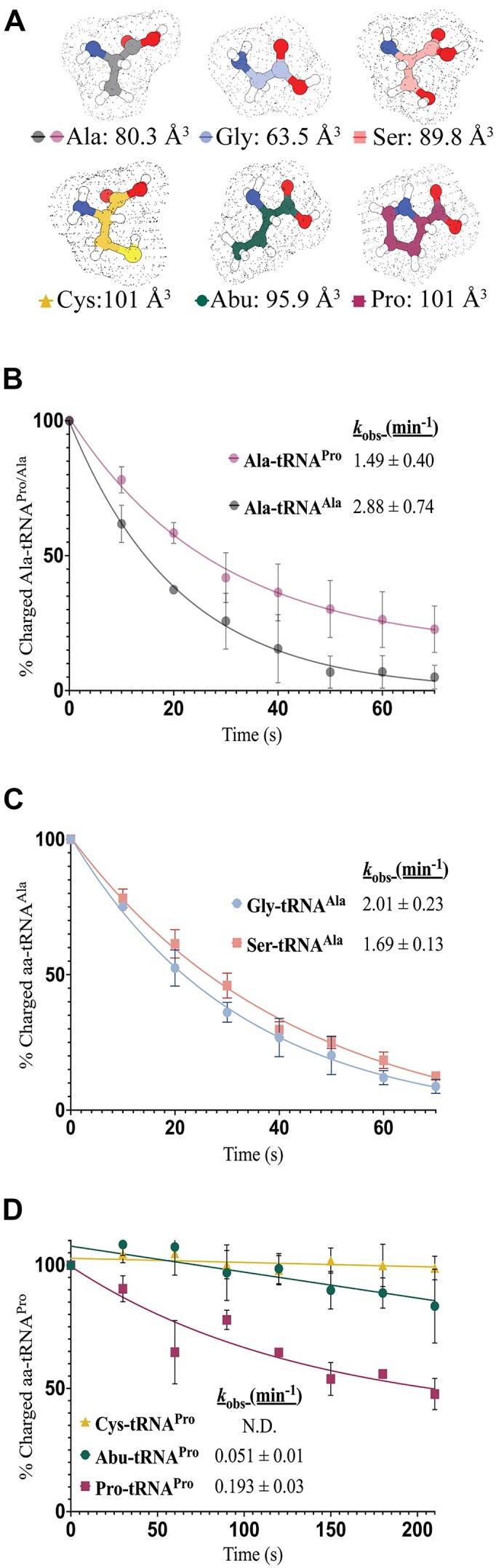
Amino acid specificity of WT *Tb* MCP1. (**A**) Structures and estimated volumes of the amino acids tested. Volumes were determined using ChimeraX and structures of free amino acids were from PubChem [48, 49]. Panels (**B**)–(**D**) display single-turnover kinetic assays conducted using 500 nM *Tb* MCP1 and 50 nM substrate at 27°C. Rate constants are reported within each plot and the standard deviation of three independent trials is indicated. (**B**) Deacylation of Ala–tRNA^Ala^ (bottom curve,•) Ala–tRNA^Pro^ (top curve,•). (**C**) Deacylation of Gly– (•) and Ser–tRNA^Ala^ (▪). (**D**) Deacylation of Cys- (▴), Abu- (•), and Pro-tRNA^Pro^ (▪).

We next probed the amino acid specificity of MCP1 by testing substrates resulting from known ARS mischarging events—AlaRS commonly mischarges tRNA^Ala^ with Ser and Gly, while ProRS can misacylate tRNA^Pro^ with Ala, Cys, and 2-aminobutyric acid (2-Abu) (Fig. 6A) [3]. Under single-turnover conditions at 27°C, MCP1 deacylated Ala–tRNA^Pro^, as well as Ser- and Gly–tRNA^Ala^ with similar efficiencies as Ala–tRNA^Ala^ in the absence of other factors (Fig. 6B,C). These data suggest that MCP1 can correct errors of mis-aminoacylation by both AlaRS and ProRS *in vitro*.

Although Ala and Abu only differ in size by a single methylene unit (80 Å^3^ versus 96 Å^3^, respectively), the catalytic pocket of MCP1 was unable to accommodate the larger Abu-tRNA^Pro^ substrate (Fig. 6A and D). Since Cys is slightly larger in volume (101 Å^3^) and more polar than Abu, Cys-tRNA^Pro^ was also not a substrate for MCP1 (Fig. 6A and D). Correctly aminoacylated Pro-tRNA^Pro^ was a substrate for deacylation, albeit with an ∼8-fold decreased efficiency compared to Ala–tRNA^Pro^ (Fig. 6D). Although Pro has the same calculated molecular volume as Cys, the limited conformational freedom of the pyrrolidine ring eliminates undesirable steric clashes that may be present with the Cys and Abu sidechains, allowing moderate deacylation.

### MCP1 rescues *Tb* AlaRS editing deficiencies

**Figure 7.**
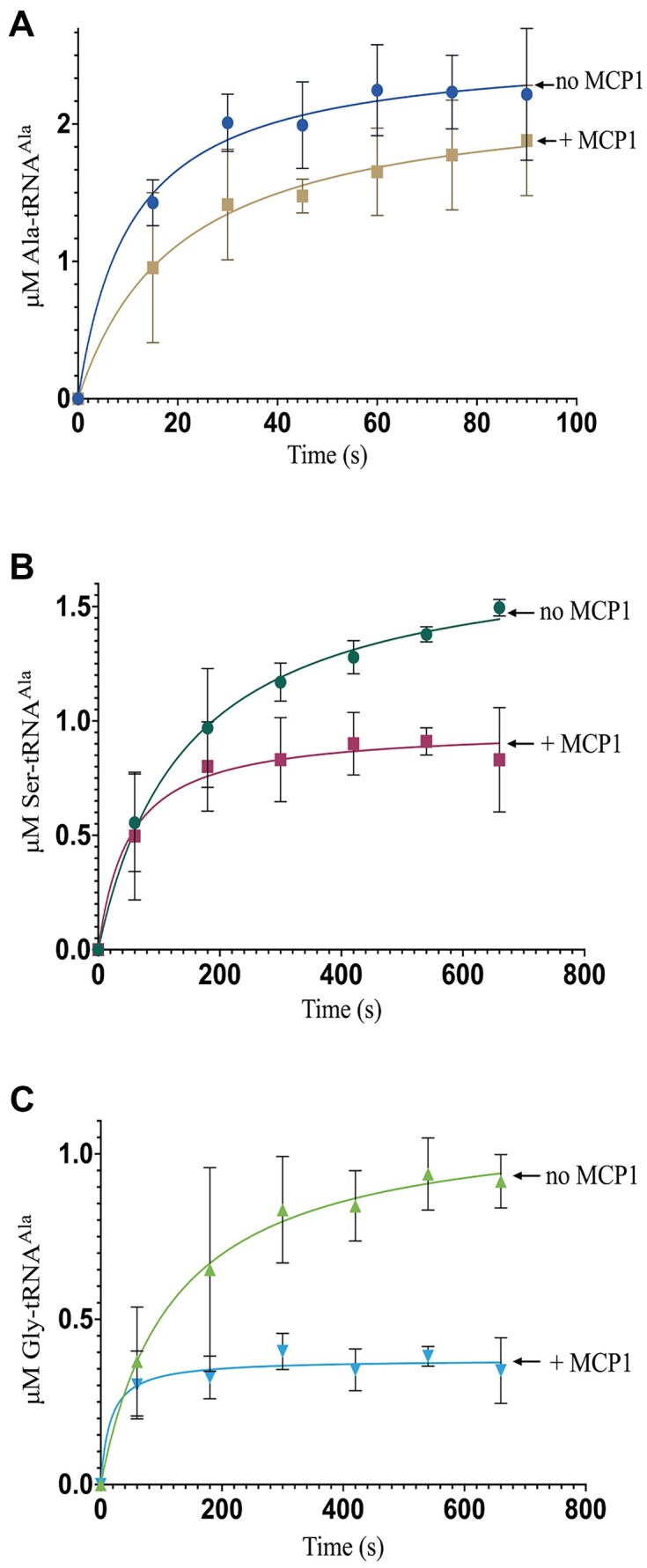
Effect of MCP1 on *Tb* AlaRS (mis)aminoacylation. (**A**) Cognate charging of 4.5 μM of [^32^P]–tRNA^Ala^ with Ala using 50 nM *Tb* AlaRS in the presence and absence of 4 μM MCP1. (**B**, **C**) Misaminoacylation of Ser–tRNA^Ala^ (**B**) and Gly–tRNA^Ala^ (**C**) with 50 nM of *Tb* AlaRS in the presence and absence of 4 μM MCP1. All assays were conducted at 27°C and contained 4.5 μM nonradiolabeled tRNA^Pro^, tRNA^Met^, and tRNA^Gln^ in addition to 4.5 μM [^32^P]–tRNA^Ala^. Error bars represent the average of three independent trials.

To mimic the cellular context of MCP1 deacylation activity more closely *in vitro*, we next conducted AlaRS aminoacylation assays in the presence and absence of MCP1, saturating levels of amino acids (Ala, Gly, or Ser), and with a tRNA pool consisting of an equimolar mixture of [^32^P]-labeled tRNA^Ala^ and nonradiolabeled uncharged competitor tRNAs (tRNA^Pro/Met/Gln^). These three tRNAs were chosen as ProRS, MetRS, and GlnRS are members of the MSC, along with AlaRS and MCP1. Under these conditions, cognate Ala–tRNA^Ala^ charging was moderately decreased in the presence of MCP1, with an ∼15% reduction in the observed plateau (Fig. 7A). Even with an intact AlaRS editing domain, *Tb* AlaRS forms an appreciable amount of Ser- and Gly–tRNA^Ala^ in the absence of MCP1 (Fig. 7B and C). Addition of MCP1 resulted in an ∼45% decrease in formation of misacylated Ser–tRNA^Ala^ and a ∼60% reduction in Gly–tRNA^Ala^ formation (Fig. 7B and C). The observed deacylation of Ala–, Gly–, and Ser–tRNA^Ala^ in the presence of excess uncharged tRNA species suggests that MCP1 prefers aa–tRNAs as binding partners.

### Enzymatic activity is conserved between *Tb* MCP1 and *Sc* Arc1p

**Figure 8.**
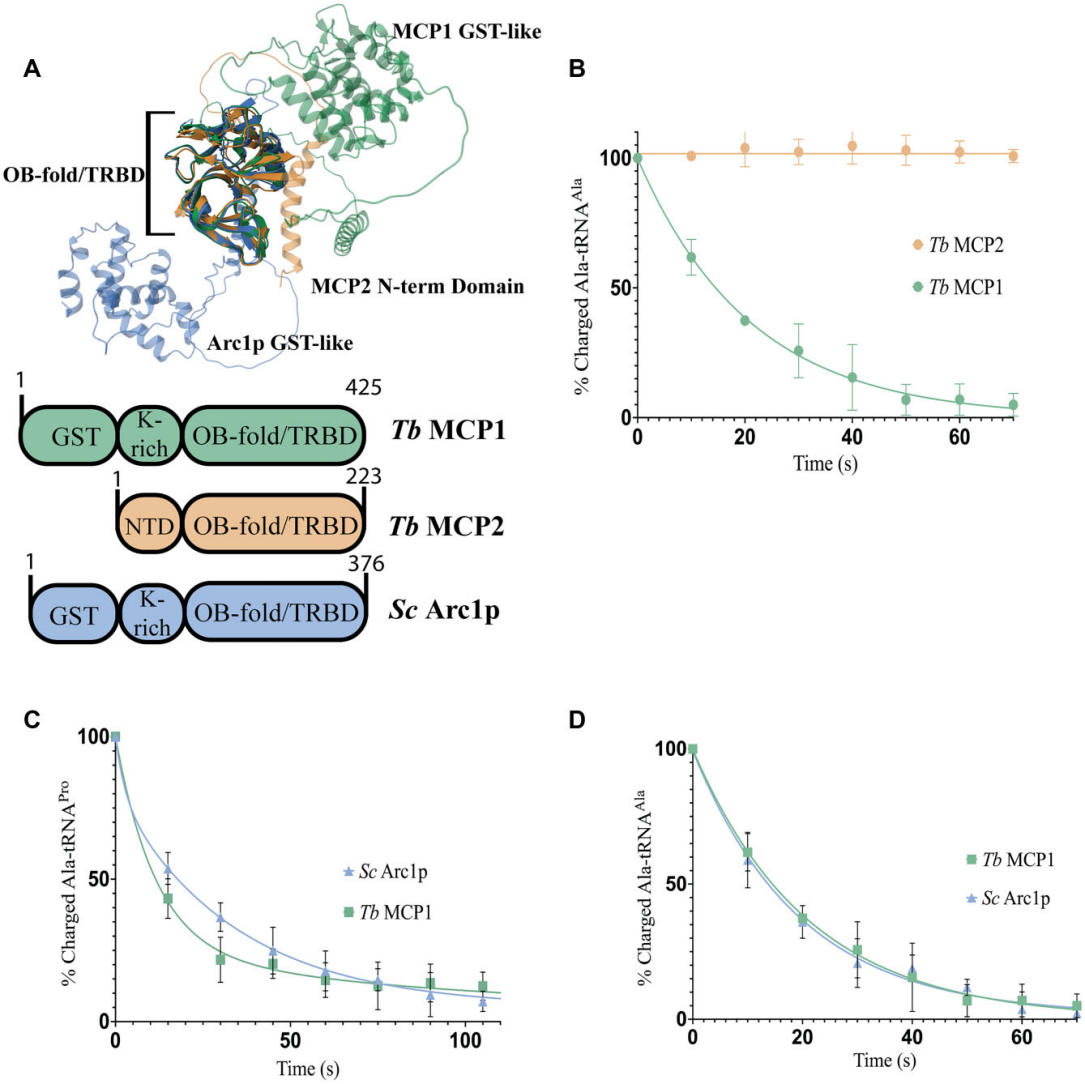
Structural models and deacylation activity of *Tb* MCP1, *Tb* MCP2, and *Sc* Arc1p. (**A**) Alphafold3 models of *Tb* MCP1 (green), *Tb* MCP2 (orange), and *Sc* Arc1p (blue) with the OB-fold domains superimposed. (**B**, **D**) Single-turnover analysis of deacylation activity using 500 nM protein and 50 nM Ala–tRNA. All assays were carried out at 27°C and error bars represent the standard deviation of three independent trials. (**B**) Comparison of *Tb* MCP1 and MCP2 Ala–tRNA^Ala^ activity. (**C**) Comparison of *Tb* MCP1 and *Sc* Arc1p Ala–tRNA^Pro^ deacylation activity. (**D**) Comparison of *Tb* MCP1 and *Sc* Arc1p Ala–tRNA^Ala^ hydrolytic activity.

Given the high sequence identity (38%) and structural similarity between the OB-folds of *Sc* Arc1p and *Tb* MCP1, as well as the conservation of K326, R331, and S335, which we showed are critical for MCP1 enzymatic activity (Figs 3 and 4), we postulated that Arc1p would also display tRNA deacylation activity. *Tb* MCP2 is also an OB-fold protein present in the *Tb* MSC and is also predicted by AlphaFold3 to have high structural similarity to the OB-fold of MCP1 (Fig. 8A). However, this protein lacks two of the three catalytic residues we identified in MCP1—a Glu residue is found in place of K326, whereas R331 is substituted with a Gly. As expected, *Tb* MCP2 did not hydrolyze Ala–tRNA^Ala^ (Fig. 8B). In contrast, *Sc* Arc1p displayed similar deacylation kinetics as *Tb* MCP1 using *Tb* Ala–tRNA^Ala^ and Ala–tRNA^Pro^ as substrates, indicating that the enzymatic activity of this family of OB-fold proteins is not species-specific (Fig. 8C and D).

## Discussion

**Figure 9.**
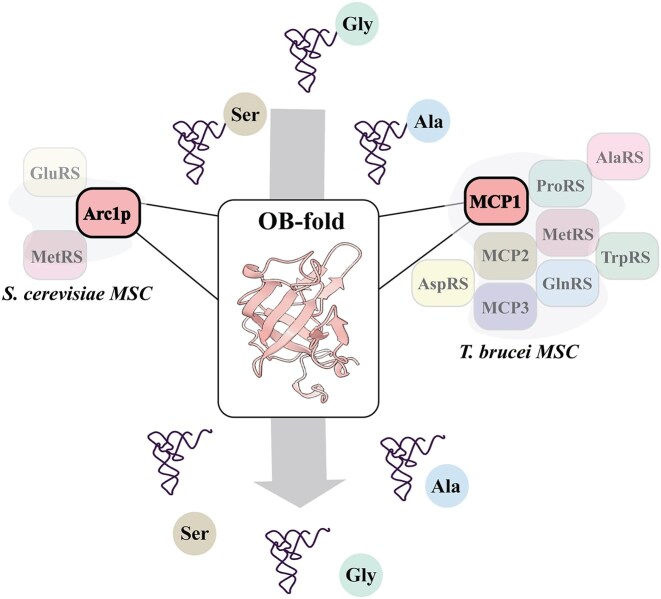
aa–tRNA proofreading by *Sc* Arc1p and *Tb* MCP1. The MSCs of *S. cerevisiae* and *T. brucei* house enzymatic OB-fold proteins, Arc1p and MCP1, respectively. Both Arc1p and MCP1 deacylate tRNAs acylated with small amino acids (Ala, Gly, Ser). While we have not demonstrated that Arc1p deacylates Gly- and Ser–tRNA, given its sequence and structural similarity to MCP1, we expect this to be the case.

In this study, we discover the unexpected catalytic activity of an ancient and well-studied nucleic acid-binding domain: the OB fold. OB folds are present in catalytic and noncatalytic proteins and bind to a range of nucleic acids: G-quadruplexes, single/double-stranded DNA, and various types of RNA, including tRNA [50–52]. According to the extended version of the Structural Classification of Proteins database, nucleic acid-binding proteins are just one superfamily within the 17 superfamilies of proteins containing OB folds [53]. OB-folds are ubiquitous in enzymes, such as inorganic pyrophosphatase and ARSs, and are often present in large catalytic complexes, but to date have been reported to only play a structural or substrate-binding role [50, 52]. To our knowledge, our study of *Tb* MCP1 and *Sc* Arc1p reveals the first example of an OB-fold family with intrinsic catalytic activity (Fig. 9).

Of the 17 sub-families within the nucleic acid-binding OB-fold superfamily, two are associated with tRNA binding: (i) the ARS anticodon-binding domain (ACBD) and (ii) the Myogenic factor (Myf) families. The ACBD OB fold is typically found in AspRS and lysyl-tRNA synthetases; other ARSs have OB folds, but they are not located within the ACBD. An X-ray crystallography co-crystal structure of *Thermus thermophilus* AspRS bound to cognate tRNA (PDB: 1EFW) shows an interaction between the anticodon stem of tRNA and the front face of the OB-fold β-barrel [24, 54]. Early tRNA-binding studies (i.e. footprinting and electrophoretic mobility shift assays) of *Ae* Trbp111, which belongs to the Myf family, concluded that this freestanding OB-fold bound with high-affinity to the outside of the elbow region of tRNAs and may facilitate tRNA folding [55–58]. In contrast, the recent X-ray crystallography-based co-crystal structure of *Ae* Trbp111 (PDB: 8VU0) revealed interaction with the CCA 3′-end of *Ec* tRNA^Ile^ using the cavity of the β-barrel [24]. The functional significance of this binding mode was unclear, although it was proposed to function as a chaperone for tRNA trafficking or alternatively, to protect the single-stranded 3′-end from degradation or nonspecific interactions [24]. Juru *et al.* also noted that “…the Trbp111 OB-fold is adaptable enough to bind both RNA and DNA tails, and may even accommodate aa–tRNAs since it does not recognize the *cis* diol and tolerates some 3′ adducts” [24]. Indeed, in the case of the Trbp111-like proteins Arc1p and MCP1 investigated here, this OB-fold binding strategy allows for aa–tRNA deacylation.

Based on assays with MCP1 domain truncations, whether standalone OB-fold domains, such as *Ae* Trbp111, function as enzymes is unclear. Deletion of both the GST domain and the lysine-rich linker region of MCP1, domains that are also present in Arc1p, significantly reduced activity but did not eliminate the ability of the TRBD to hydrolyze Ala–tRNA^Ala^. This suggests regions other than the OB-fold-containing domain are required for optimal tRNA deacylation, likely by contributing to binding stability. Studies with Arc1p showed that deletion of the N-terminal GST-like domain alone surprisingly increased tRNA binding affinity, but additional deletion of the entire linker domain abolished binding [24]. Deletion of the GST-like domain from MCP1 reduced deacylation activity ∼3-fold, which we attribute to the inability of ΔGST-MCP1 to dimerize as supported by our MP analyses showing: (i) the ΔGST-MCP1 was unable to oligomerize and (ii) the addition of tRNA resulted in a two-fold increase in the relative abundance of WT MCP1 dimer. Whether two copies of MCP1 independently associate with different regions of the tRNA and then use GST-mediated dimerization to stabilize the complex or for dissociation, requires further structural investigation. Mutation of K326, R331, and S335 to Ala in Tb MCP1 resulted in >100-fold decrease in the single-turnover rate constants. The effect of these positions on enzymatic activity is greater than their impact on tRNA binding—the same mutations made in the context of Arc1p only increased the Kd for tRNAIle ∼3–9-fold [24].

All eukaryotic MSCs reported to date house at least one non-ARS OB-fold containing factor in the Myf family (Trbp111-like), except in *Caenorhabditis elegans* where the Trbp111-like domain is fused to the C-terminus of MetRS; the functional significance of this observation and whether they all display tRNA deacylation activity is still unknown [28, 30]. In yeast, Arc1p proofreading could protect the cell against tRNA^Pro^ mischarging events as *Sc* ProRS encodes a defunct N-terminal INS-like editing domain [59]. In addition, no other free-standing INS superfamily members such as the known Ala– or Cys–tRNA^Pro^*trans*-editing domains (ProXp-ala, MCP3, YbaK, etc) are encoded in the *S. cerevisiae* genome [3, 60]. In *T. brucei*, MCP1 represents one of three possible strategies to proofread tRNA^Pro^, as MCP3 and a ProXp-ala domain fused to ProRS also display robust Ala–tRNA^Pro^ hydrolytic activity [31]. *Trypanosoma brucei* parasites navigate between two hosts during their lifecycle and undergo unique metabolic changes that include significant fluctuation in the ratio of free Ala and Pro, which may explain the need for redundant tRNA^Pro^ fidelity mechanisms [61, 62]. With regards to AlaRS aminoacylation errors, *S. cerevisiae* and *T. brucei* are equally equipped to maintain accurate translation. In addition to Arc1p/MCP1, both genomes encode a Ser/Gly–tRNA^Ala^ editing domain within AlaRS, a freestanding AlaXp domain for Ser–tRNA^Ala^ hydrolysis, and a D-aminoacyl–tRNA deacylase for Gly–tRNA^Ala^ proofreading [63, 64]. Whether the deacylation activity of Arc1p/MCP1 is one of several physiological mechanisms that keep AlaRS in check remains an open question—our *in vitro* aminoacylation assays suggest this is probable, as *Tb* AlaRS misacylated tRNA^Ala^ despite the presence of an appended editing domain.

Our results show that MCP1 alone promiscuously deacylates Ala–tRNA^Ala^ (Fig. 1B) but fails to significantly prevent aminoacylation by AlaRS (Fig. 7). These data strengthen the notion that MSCs exist, at least in part, to regulate the canonical and noncanonical activities of their components, as previously proposed based on studies with MCP3 [64]. Previous studies also showed that *Sc* Arc1p could form a stable complex with various tRNA species, with a preference for Glu, Met, Phe, Lys, Arg, Pro, Ala, and Ser tRNA isoacceptors [19]. Based on the amino acid specificity and promiscuity of the freestanding MCP1/Arc1p proteins demonstrated in this work, we propose that in addition to previously proposed roles for the *Sc* MSC [19–23, 27, 65], integration of Arc1p/MCP1 into MSCs protects the correctly aminoacylated tRNA pool from deacylation.

Studies with Arc1p established that it is not essential for yeast viability at normal growth temperatures (30°C). However, at temperatures above and below 30°C, *Sc* Arc1p knock out results in a significantly slower growth phenotype [22]. *Sc* Arc1p is also implicated to play a role in tRNA trafficking. Knock-out of both Arc1p and tRNA nuclear-export factor Los1p is lethal under nutrient-rich growth conditions—Los1p deletion alone does not impact yeast growth [66]. Interestingly, the lethality of ΔArc1pΔLos1p can be rescued by overexpression of eEF1-α, a factor that binds to aa–tRNAs [66]. Rescue of the phenotype may be due to eEF1-α sequestration of otherwise lethal mis-aminoacylated tRNAs. Whether the surprising aa–tRNA proofreading activity of Arc1p reported here contributes to the previously reported phenotypes remains to be established.

## Supplementary Material

gkaf328_Supplemental_File

## Data Availability

All data described are contained within the article. Requests to access the datasets should be directed to K.M.-F. musier-forsyth.1@osu.edu.
