# Supplementary material for: Unexpected enzymatic function of an ancient nucleic acid-binding fold
Source: Nucleic Acids Res. 2025 Apr 25;53(8):gkaf328. doi: 10.1093/nar/gkaf328 (PMC12021450; doi:10.1093/nar/gkaf328)
Supplement: gkaf328_Supplemental_File [file gkaf328_supplemental_file.pdf]

Unexpected enzymatic function of an ancient nucleic acid-binding fold

Supplementary Material

Rylan R. Watkins[1], Stella Bockelman[1, †], Anna Vradi[1,†], Kaylee Grabarkewitz[1], Alexa Pyun[1], Josephine Stark[1], Vicki H. Wysocki[1], Juan D. Alfonzo[2], and Karin Musier-Forsyth[1*]

[1]Department of Chemistry and Biochemistry, Center for RNA Biology, Ohio State University, Columbus, OH

[2]Department of Molecular Biology, Cell Biology and Biochemistry, The Brown RNA Center, Brown University, Providence, RI

[†]Authors contributed equally to this work.

*To whom correspondence should be addressed: musier-forsyth.1@osu.edu

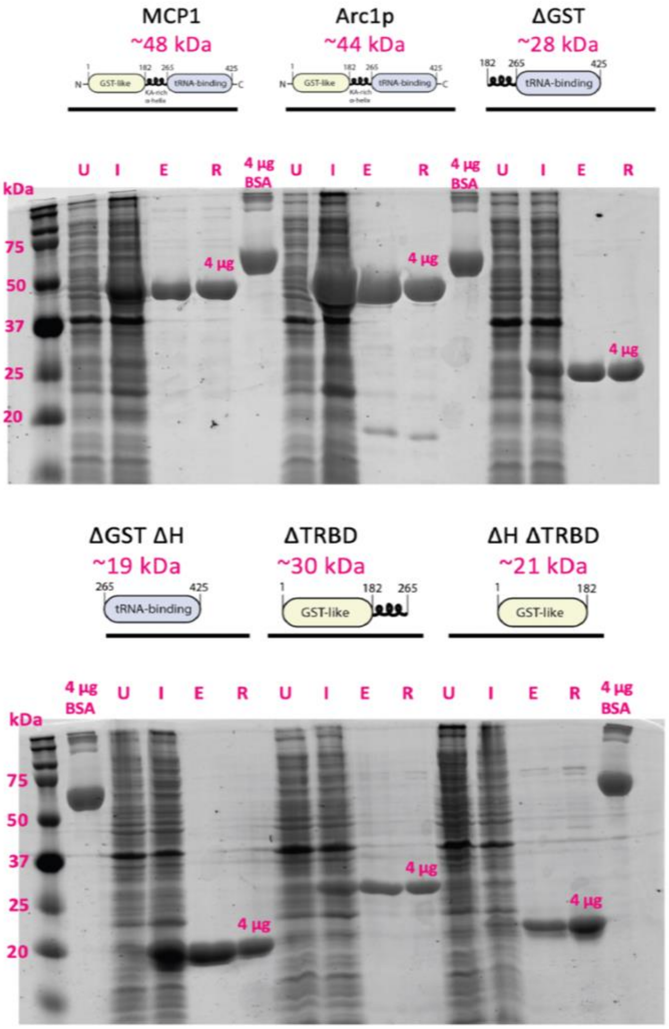

Supplemental Fig 1. SDS-PAGE (12%) analysis of recombinantly-expressed and purified proteins used in this study. *Top gel: Tb* MCP1, *Sc* Arc1p, and *Tb* MCP1 lacking the GST domain (ΔGST). *Bottom gel: Tb* MCP1 lacking: the GST and linker domains (ΔGSTΔH), the TRBD domain (ΔTRBD), or the linker domain and TRBD (ΔHΔTRBD). U = uninduced bacterial culture; I = IPTG-induced culture; E = Ni^{2+} affinity-column elution; R = final purified protein used for kinetic assays (4 µg). A 4 µg BSA standard was included on each gel for reference. Gel is stained with Coomassie G250 and imaged with Typhoon RGB imager using Cy5 excitation/emission channel.
